# Efficient removal of As(V) from aqueous media by magnetic nanoparticles prepared with Iron-containing water treatment residuals

**DOI:** 10.1038/s41598-020-65840-1

**Published:** 2020-06-09

**Authors:** Huiping Zeng, Longxue Zhai, Tongda Qiao, Yaping Yu, Jie Zhang, Dong Li

**Affiliations:** 1 Key Laboratory of Water Quality Science and Water Environment Recovery Engineering, Beijing University of Technology, Beijing, 100124 China; 2 State Key Laboratory of Urban Water Resource and Environment, Harbin Institute of Technology, Harbin, 150090 China

**Keywords:** Environmental sciences, Chemistry, Materials science, Nanoscience and technology

## Abstract

Two types of magnetic nanoparticles prepared with chemical agents (cMNP) and iron-containing sludge (iMNP), respectively, were synthesized by co-precipitation process and used to remove arsenate [As(V)] from water. The synthesized magnetic adsorbents were characterized by XRD, XPS, TEM, BET, VSM and FTIR. The adsorbents iMNP and cMNP were both mainly γ-Fe_2_O_3_ in nanoscale particles with the saturation magnetization of 35.5 and 69.0 emu/g respectively and could be easily separated from water with a simple hand-held magnet in 2 minutes. At pH 6.6, over 90% of As(V), about 400 μg/L, could be removed by both adsorbents (0.2 g/L) within 60 min. The adsorption isotherm of both fabricated materials could be better described by the Langmuir adsorption isotherm model than the Freundlich’s, In addition, the adsorption kinetics of both adsorbents described well by the pseudo-second order model revealed that the intraparticle diffusion was not just the only rate controlling step in adsorption process. With the larger maximum As(V) adsorption capacity of iMNP (12.74 mg/g), compared with that of cMNP (11.76 mg/g), the iMNP could be regarded as an environmentally friendly substitute for the traditional magnetic nanoparticles prepared with chemical agents.

## Introduction

Arsenic, the twentieth richest element in the earth’s crust, is widely dispersed into water due to mining, natural weathering process, dissolution of minerals, agricultural pesticides and other natural and anthropogenic activities^1,2^. Long-term use of such arsenic contaminated water during human’s daily life, such as drinking, cooking and irrigation, would pose danger to human health, including skin, lung cancers, cardiovascular diseases, non-pitting swelling and other diseases^3,4^. Consequently, arsenic contamination of ground water has drawn attention all over the world and the WHO (World Health Organization) and EPA (Environmental Protection Agency) regulations set 10 ug/L as the maximum contaminate level of arsenic in drinking water. Arsenic in natural water sources exist predominately in two forms of arsenite (As(III)) and arsenate (As(V)). However, in groundwater, trivalent arsenic is the main existing form of arsenic, and the toxicity of arsenite is about more 25~60 times than that of arsenate. Furthermore, it is easier to remove arsenate from water than arsenite^5–7^. For these reasons, arsenite is always oxidized to arsenate by oxidation technologies prior to remove them from water. Hence, how to remove arsenate from aqueous media will be particularly investigated in this study.

In recent years, adsorption is considered one of the most competitive methods for arsenic remediation from water due to its high efficiency, economy, and simple operation^8^. There are various adsorbents made for arsenic removal including natural and composite materials, in which nanoadsorbent have drawn tremendous attention, as it have large surface area and a number of surface active sites^9^. Among numerous nanomaterials, magnetic nanomaterial is a kind of pretty special and promising material due to its directional movement in the magnetic field, with which it can be separated from solution after adsorption via a simple magnet. Particularly, superparamagnetic nanomaterial can be rapidly magnetized when applied a magnetic field and rapidly demagnetized when the external magnetic field is removed, exhibiting zero remanence and zero coercivity. Based on the previous research, magnetite (Fe_3_O_4_) and maghemite (γ-Fe_2_O_3_) nanoparticles are two kinds of magnetic nanoparticles, which are the most widely studied because of its cost-effective, environmentally friendly and stable properties. Jiang *et al*.^10^ prepared maghemite nanoparticles via a chemical co-precipitation method and investigated its properties for Chromium (VI) remediation. Shan *et al*.^11^ showed arsenite (As(III)) could be effectively removed by Mag-Fe-Mn, where γ-Fe_2_O_3_ was employed as the magnetic core and the Fe-Mn binary oxide as the coating materials. Mamun *et al*.^12^ investigated the adsorption of arsenic on magnetic iron oxide nanoparticles (Fe_3_O_4_) coated with humic acid. In this study, the primarily concern is Fe_3_O_4_ and γ-Fe_2_O_3_ nanoparticles.

Drinking water purification process, using groundwater as the source, always produces large amount of sludge as waste^13^. Most of such sludge consists of amorphous masses of iron and aluminum hydroxides and it also contains mineral and humic matters removed from the raw water, along with the residuals of coagulant agents used in the drinking water treatment process^14^. It is a common practice to dispose water treatment residuals (WTRs) in landfill, however, it takes up much space and investment. Especially for the dispose of iron-containing water treatment residuals that collected from backwash wastewater from the biofilter containing a certain amount of iron and numerous impurities, such as aluminum, sand, and broken filter material, the cost of landfill becomes higher, because iron sludge has to be dewatered through flocculation, coagulation, and filter pressing prior to landfill disposal^15^. To protect environment and economizing resource, the reuse of WTRs has drawn worldwide attention of researches, and some relative research works has been made. Elkhatib *et al*.^16^ investigated how the mobility and phytoavailability of Cd and Pb in biosolid-amended soils influenced by the nanoparticles of WTRs. Chiang *et al*.^17^ studied the WTRs adsorption capacities and applications for multi-heavy metals.

In this study, our goal of reusing WTRs is to contribute to building an environmentally friendly and resource-efficient community, providing researchers with a more economical substitute for magnetic nanoparticles prepared with chemical agents. This study utilizes simple co-precipitation methods to fabricate magnetic nanoparticles and compare the difference among magnetic nanoparticles prepared with iron-containing WTRs and chemical agents respectively based on the adsorption capacities for arsenate and its properties.

## Materials and Methods

### Materials

The iron sludge was obtained by precipitating the backwashing wastewater collected from a groundwater plant in Harbin, Heilongjiang province of Northeast China. After the wastewater precipitated for several hours, the supernate was discarded and the settled yellowish brown iron sludge on the bottom was naturally air-dried. The sludge was ground with a mortar, sieved with a 100 mesh sieve, and then stored in a desiccator. The main element components of the iron sludge can be seen in our previous study^18^.

As(V) stock solutions were synthesized by dissolving Na_2_HAsO_4_·7H_2_O of analytical grade with ultrapure water (resistivity >18.0 MΩ cm) from an integral water purification system (Milli-Q, Millipore, Billerica, MA, USA).

**Scheme 1:**
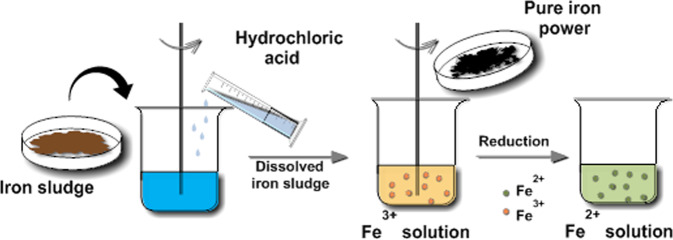
Process of Fe^3+^ and Fe^2+^solution prepared.

Fe^3+^ stock solutions were obtained by dissolving iron sludge into a certain amount of hydrochloric acid, and then a part of Fe^3+^ solutions were taken out to prepared Fe^2+^ stock solutions by reduction with pure iron powder (Scheme 1).

Potassium borohydride, Pure iron powder, sodium hydroxide, thiourea used in the experiment were of guarantee grade, Ferrous sulfate heptahydrate, ferric chloride, hydrochloric acid and Na_2_HAsO_4_·7H_2_O were of analytical grade, were purchased from Tianjin Fuchen Chemical Reagents Factory (Tianjin, China). The solutions were prepared with deionized or ultrapure water according to need.

### Adsorbent preparation

**Scheme 2:**
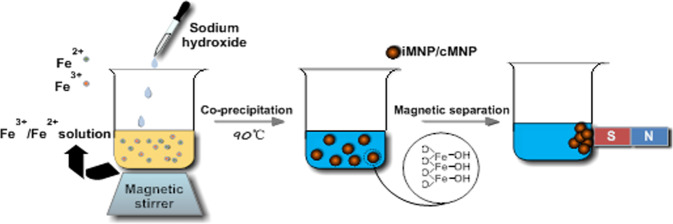
Steps involved in the synthesis of the iMNP and cMNP.

The adsorbent prepared with iron sludge (iMNP), was fabricated via a chemical co-precipitation process. Specifically, the concentrations of Fe^3+^ and Fe^2+^ solutions were determined by employing phenanthroline spectrophotometry, and were adjusted to what we need (i.e. 0.36 M Fe^2+^ and 0.28 M Fe^3+^) with deionized water. 50 mL 0.72 M Fe^2+^ and 50 mL 0.56 M Fe^3+^ solutions were mixed into a 500-mL beaker and heated with magnetic stirring until the temperature reached to 90°C. At this temperature, 10 M NaOH solution was added into the reaction mixture until the pH reached around 11 when the black precipitate formed. The mixture was kept at 90 ± 1°C and stirred for another 30 min. After cooled to the room temperature, the precipitate was washed with deionized water and then collected with a simple magnet, the washing procedure was repeated several times (Scheme 2). Finally, the adsorbent was dried in a vacuum oven at 70°C for 2 hours and stored in a desiccator for further use.

For comparative purpose, the procedures to prepare another adsorbent named cMNP were similar to those for iMNP. The difference was that the iron solutions were from FeSO_4_·7H_2_O and FeCl_3_·6H_2_O rather than iron sludge.

### Adsorbent characterization

Hysteresis loops of the adsorbents were measured at room temperature by using a Vibrating sample magnetometer (VSM, Versalab, Quantum Design, USA). To better characterize the surface morphology and size of the magnetic nanoparticles, the pretreatment of Transmission electron microscopy (TEM) need to be done and is presented as followed: the mixture of magnetic material powder and ethanol was added to the centrifuge tube for sonicating 15 min prior to dipping onto a copper substrate grids, analyzed using a JEM 1200EX instrument operated at 120 kV after the ethanol was volatilized. The adsorbent constituents were determined by X-ray diffraction (XRD, Bruker D8 Advance, Germany) with Co Kα radiation (l = 1.79026 A) operated at a 2θ range of 10°~90°, and the operated voltage, current and Scanning speed were 40 kV, 40 mA and 6°/min, respectively. Surface area of the prepared adsorbents were measured through N_2_ adsorption/ desorption at 77.3 K with Brunauer—Emmett—Teller (BET, ASAP 2460, Micromeritics, USA). The zero charge point (pH_pzc_) is determined by the drift method^18^. At different initial pH values (4.0−10.0), a dose of adsorbents (0.05 g) was added into a solution of 100 mL with 0.1 M NaNO_3_ and mixed for 24 h, and the values of final solution pH were measured. Afterwards, a curve illustrating the relationship between ΔpH and initial pH was plotted and went through the x-coordinate, where the pH_pzc_ is equal to ΔpH = 0. The surface functional group of adsorbents that before and after adsorption of As(V) were analyzed by FTIR (Nicolet iS10 FTIR spectrometer, USA).

### Adsorption experiments

All the glassware and bottles vials used in adsorption experiments were pretreated by soaking in 10% nitric acid solution overnight prior to being used. The concentrations of a series of standard and working solutions were prepared by dilution of the stock solution As(V) with concentration of 1 g/L.

Adsorption kinetic experiments of As(V) were first conducted to obtain the contact time required to attain equilibrium. 100 mg of iMNP or cMNP was dispersed into 500 mL of As(V) solution (initial pH 6.6 ± 0.1) with concentration of 400 μg/L in a 1 L polyethylene plastic bottle and sonicated for 2 min. Then the bottles were shaken at 200 rpm under 25°C in a thermostatic orbit shaker.

**Scheme 3:**
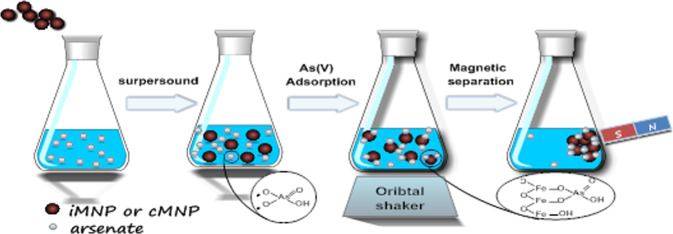
Arsenate (As(V)) removal process.

Adsorption isotherms experiments were carried out in 250 mL conical flasks with 50 mL As(V) solutions as adsorbate and 10 mg of iMNP or cMNP as adsorbent (Scheme 3). The flasks were sonicated for 2 min and then shaken in a thermostatic orbit shaker at 200 rpm for sufficient time to reach equilibrium. The test was conducted under 25 °C with a pH 6.6 ± 0.1. The adsorption isotherms of iMNP and cMNP were acquired by varying initial As(V) concentration from 1 to 10 mg/L and adsorbent dosage of 0.2 g/L. All adsorption experiments were in triplicate. After the adsorption reached equilibrium, the adsorbent with adsorbed As(V) was separated by a hand-held magnet, and the residual As(V) in the solution was measured with atomic fluorescence spectrophotometer (AFS-8230, Beijing Jitian instrument Co. Ltd.). The amount of adsorbed As(V) on the adsorbent and the percent of As(V) removal were calculated by using the following equations, respectively.

1 $${q}_{t}=\frac{({c}_{0}-{c}_{t})V}{M}$$

2 $$\varepsilon ({\rm{ \% }})=\frac{({c}_{0}-{c}_{t})}{{c}_{0}}\times 100$$

where q_t_ is the amount of As(V) adsorbed (mg/g) at time t; C_0_ and C_t_ is the initial and remaining concentration of As(V) in solution (mg/L) respectively; V is the volume of the solution (L) and M is the amount of adsorbent (g).

## Results and Disscussion

### Characterization of the adsorbents

**Figure 1:**
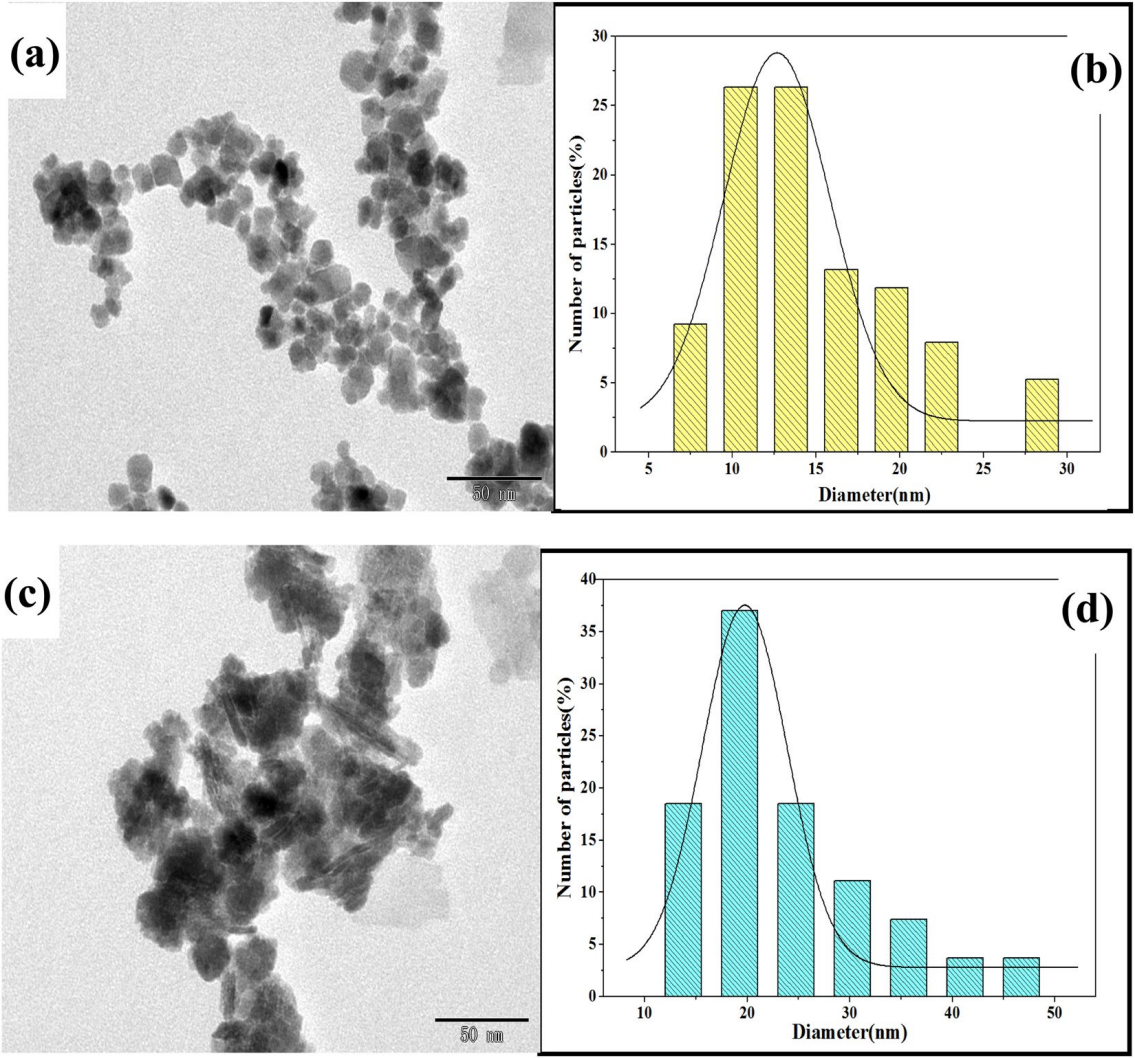
TEM micrograph of (**a**) cMNP and (**b**) histogram of their particle size distribution. (**c**) TEM micrograph of iMNP and (**d**) histogram of their particle size distribution.

As the TEM images shown in Fig. 1, one can observe that the particles of iMNP with an irregular morphology, which was larger than cMNP that were quasi-spherical in shape with the sizes in the nanoscale. This similar situation could be found in previous study^15^, they demonstrated that due to the presence of SiO_2_ synthesized by the polycondensation between silicate and Si hydroxyl groups, which were formed by quartz dissolved under alkaline condition, resulted in the grain size increases along with the particles aggregating. And SiO_2_ was presented exactly in the particle of iMNP, which may be the main reason that the particle size of iMNP (Fig. 1c) is larger than that of cMNP (Fig. 1a). Here, the nanomeasurer software is utilized to measure the diameter of particles in the TEM micrographs to obtain their corresponding size distribution (Fig. 1b,d): in terms of iMNP, the particle size ranges from 14.3 nm to 45.1 nm with the average diameter size at (23.5 ± 8.2) nm; as for the particles of cMNP, the size varies from 7 nm to 29 nm with an average diameter at (13.8 ± 6.3) nm.

**Figure 2:**
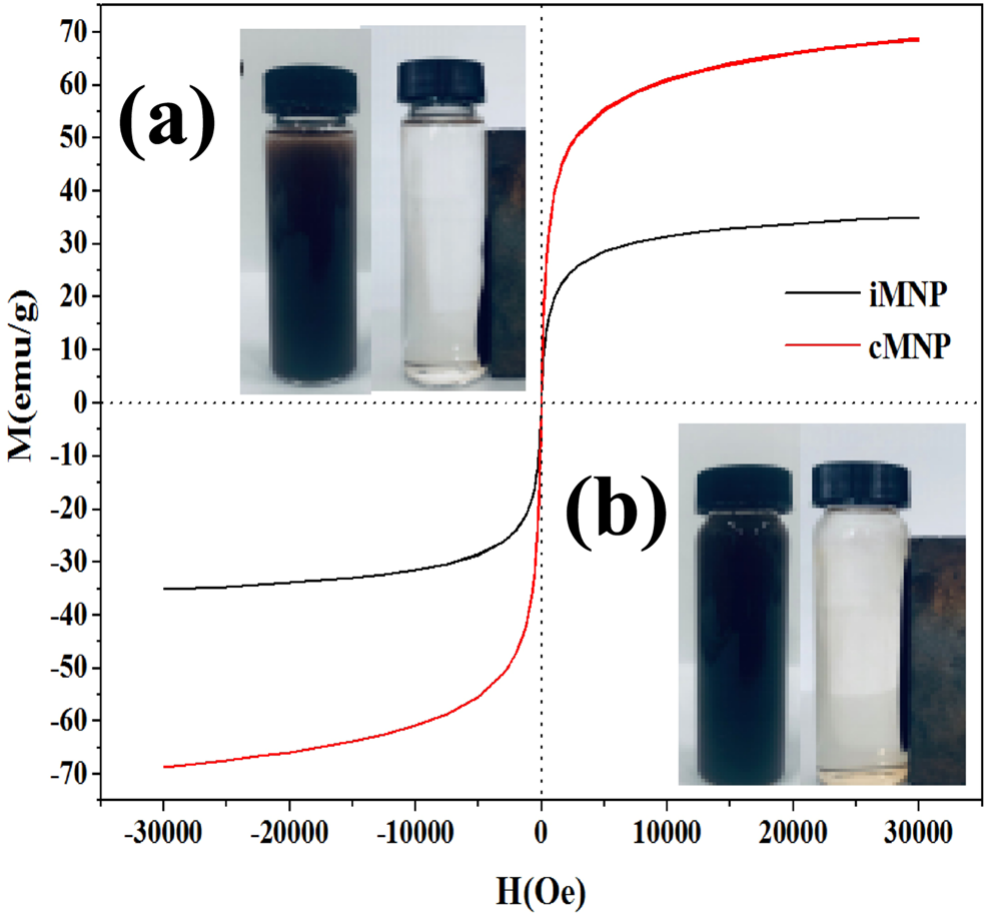
Room temperaturee magnetization curves of iMNP and cMNP and solutions before and after magnetic separation of (**a**) iMNP and (**b**) cMNP.

**Figure 3:**
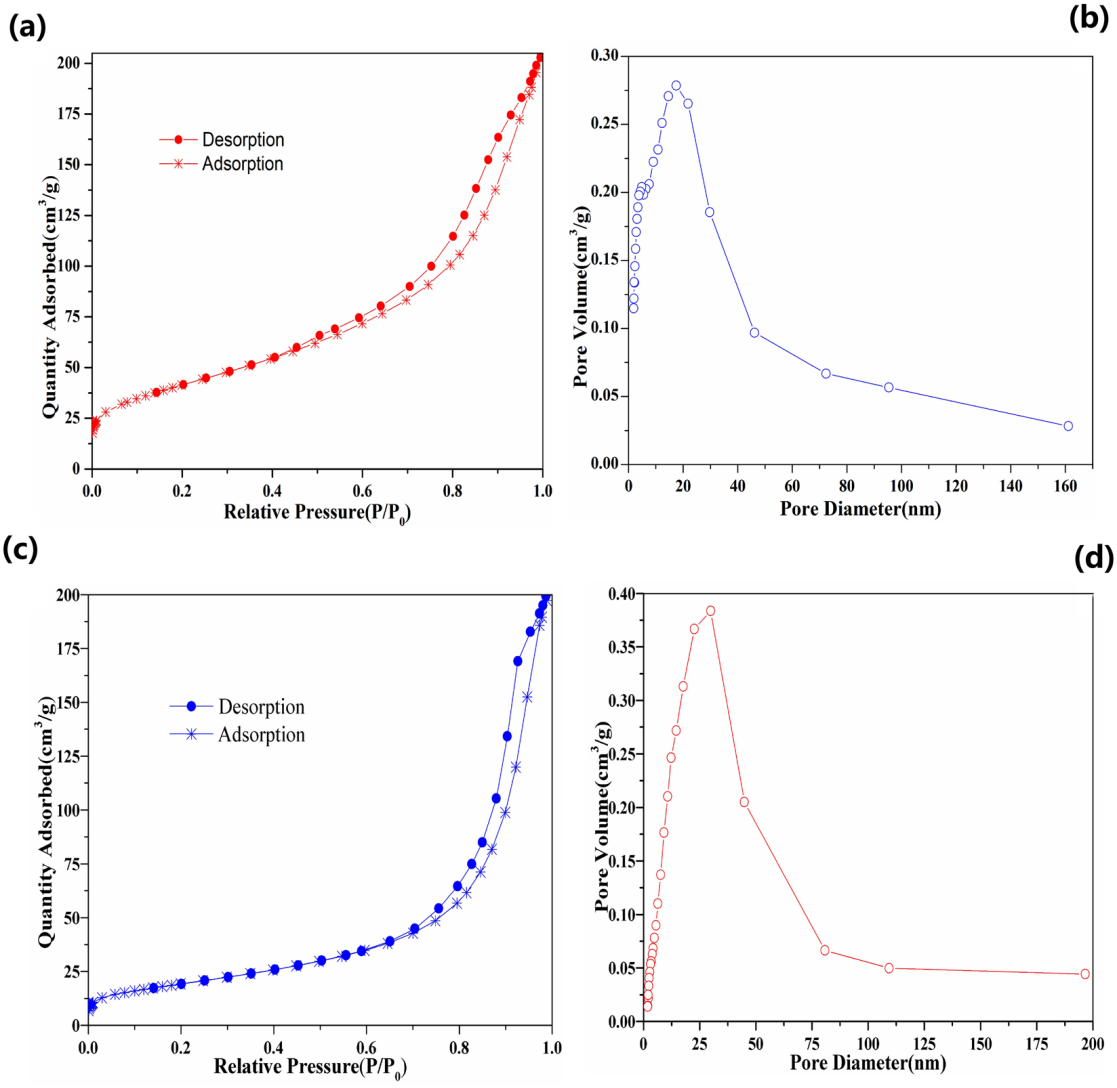
N_2_ adsorption-desorption isotherm liner plots of (**a**) iMNP and (**c**) cMNP, BJH pore size distribution of (**b**) iMNP and (**d**) cMNP.

**Figure 4:**
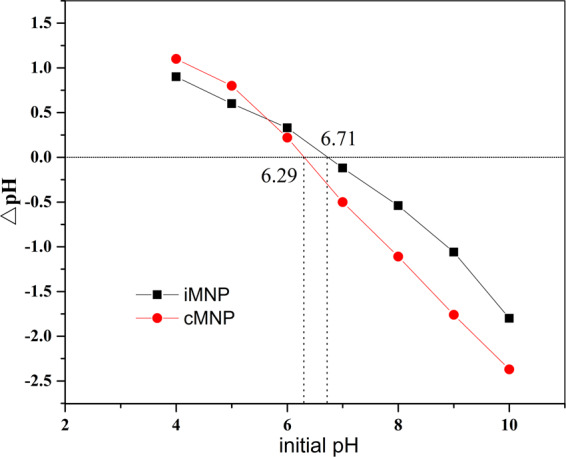
The determination of adsorbents’ pH_pzc_ based on the plots of △pH -initial pH.

The hysteresis curves of iMNP and cMNP (Fig. 2) demonstrated that both adsorbents were superparamagnetic, because there is no coercivity or remanence displayed in the curves. It can also be observed that the saturation magnetization of cMNP was 69.0 emu/g, this value decreased almost a half for iMNP (35.5 emu/g), which may be resulted from poor crystallinity caused by the non-magnetic impurities and low content of γ-Fe_2_O_3_ in unit mass of magnetic nanoparticles^19^. Despite the observed reduction of magnetization, it still be higher than that previously reported^18,20^, so the iMNP could be easily separated from the solution with a simple hand-held magnet in 2 minutes (Fig. 2). The BET surface area of iMNP and cMNP were 145.5 m^2^/g and 70.3 m^2^/g, respectively. According to the IUPAC classification, the N_2_ adsorption and desorption isotherms of both adsorbents (Fig. 3a,c) could be classified into type IV isotherms with hysteresis loops of type H3. Additionally, the mean pore diameter of iMNP and cMNP obtained from BJH pore size distribution plots (Fig. 3b,d) are about 7.8 nm and 14.4 nm, respectively. These results above illustrated that both adsorbents are mesoporous materials. As shown in Fig. 4, the isoelectric point of γ-Fe_2_O_3_ in the presence of impurities shifted to higher pH (6.71).

**Figure 5:**
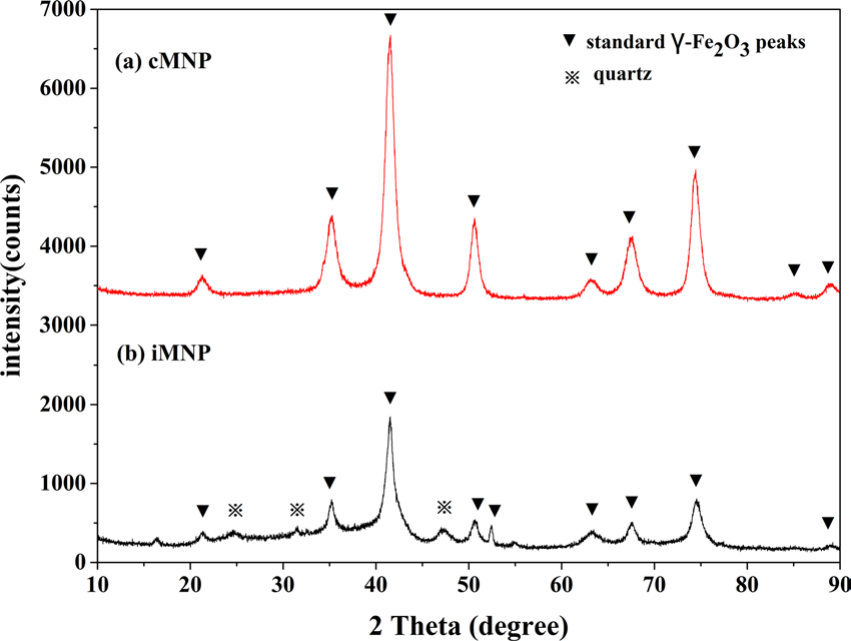
XRD pattern of iMNP and cMNP.

**Figure 6:**
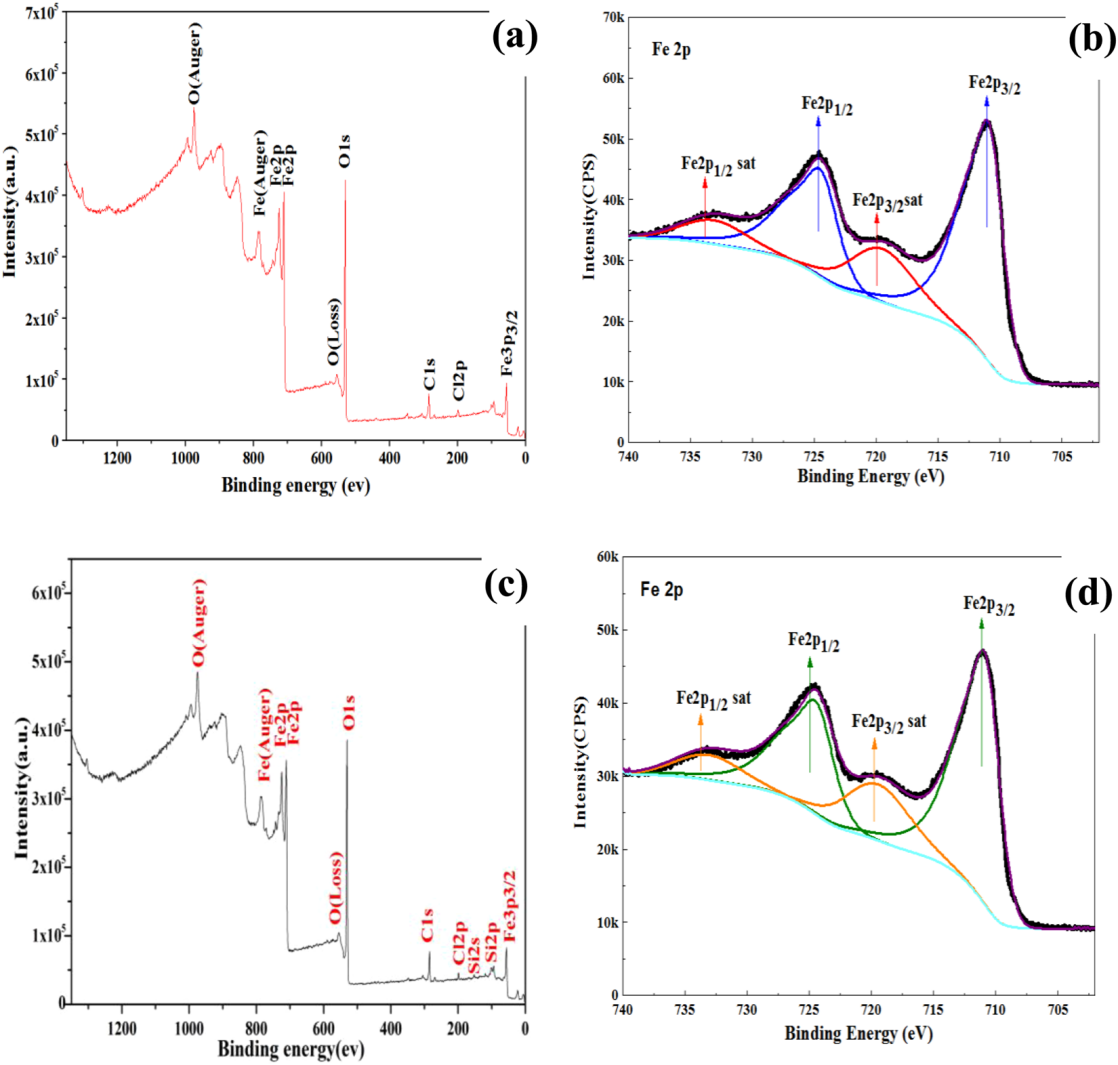
(**a**) XPS wide scan spectra and (**b**) XPS core-level spectra at Fe2p of cMNP. (**c**) XPS wide scan spetra and (**d**) XPS core-level spectra at Fe2p of iMNP.

XRD patterns of iMNP and cMNP as shown in Fig. 5 were fitted in that of standard γ-Fe_2_O_3_ (maghemite, PDF#39–1346). Compared to the XRD pattern of cMNP (Fig. 5a), apart from the crystalline γ-Fe_2_O_3_, there are also quartz phase and some broad and low intensity peaks in the pattern of iMNP (Fig. 5b), indicating that some impurities in iron sludge were not removed in the process of preparation. Since the XRD pattern of Fe_3_O_4_ and γ-Fe_2_O_3_ are quite similar, XPS was used to further confirm the main crystalline of the two prepared adsorbents. The XPS spectrums are shown in Fig. 6, the element composition of iMNP and cMNP was similar, whereas one can be observed that small amounts of Si is also presented in iMNP, which was in agreement with the result of the XRD. The binding energies of 529.9, 284.5, 198.2, 152.3, 102.2 and 56 eV were in accordance with O1s, C1s, Cl2p, Si2s, Si2p and Fe3p3/2 peaks, respectively. High energy resolution scans of Fe2p transitions are shown in Fig. 6. Binding energy of Fe2p3/2 and Fe2p1/2 were 711.0 and 724.6 eV respectively, and their corresponding satellite peaks at 719.5 and 733.1 eV were characteristic for maghemite. The separations between the satellite peaks and their associated main peaks are 8.5 eV. These results are similar to the values of maghemite particles reported previously^21–23^. The most important spectral parameters values obtained via curves-fitting presented in Table 1, indicating that the main components of iMNP and cMNP are the same. Therefore, the analysis of the curves-fitting for the Fe2p peaks suggests again the main crystalline of the prepared materials were both γ-Fe_2_O_3_.

**Table 1:** Results of the Fe2p curve fit.

	iMNP	cMNP
BE 2p_3/2_ (eV)	711	711
FWHM 2p_3/2_ (eV)	2.8	2.8
BE 2p_1/2_-2p_3/2_ (eV)	13.6	13.6
FWHM 2p_1/2_ (eV)	3.35	3.35
2p_1/2_/2p_3/2_ intensity ration	0.51	0.51
2p_3/2_ satellite shift (eV)	8.5	8.5
2p_3/2_ satellite intensity	30203.6	33831.7
Fe^2+^ proportion (%)	—	—

**Figure 7:**
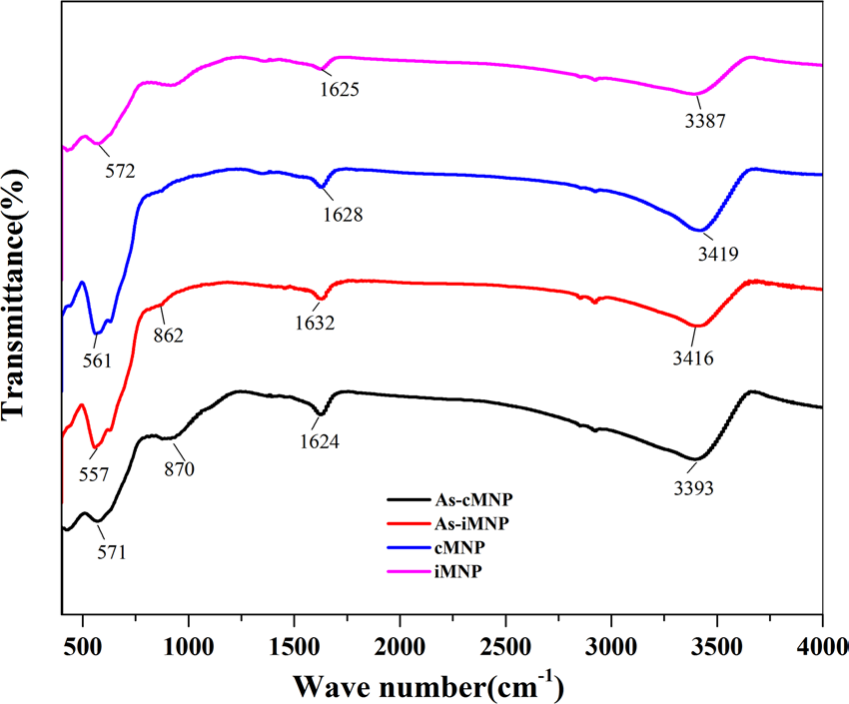
FT-IR spectrum of both adsorbents before and after adsorption of As(V).

**Figure 8:**
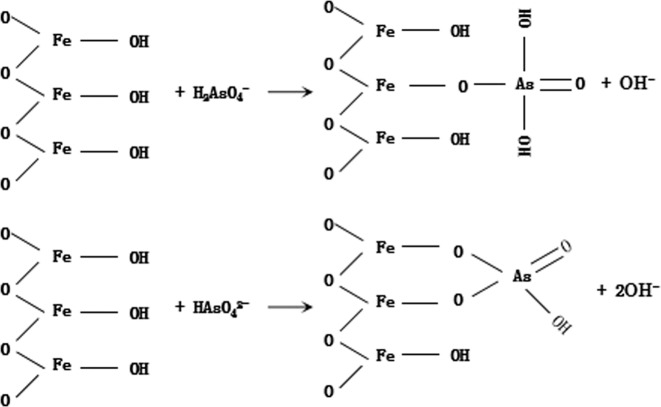
Mechanism of adsorption As(V) on the iron oxides.

FT-IR spectrum (Fig. 7) of both adsorbents (iMNP and cMNP) before and after adsorption of As(V) was used to confirm the mechanism (Fig. 8) of the adsorption As(V) on the both adsorbents. After adsorption As(V), for iMNP, the peaks at 572 cm^−1^ assigned to Fe-O stretching vibration was shifted from 572 to 557 cm^−1^, the OH–stretching and OH–bending shifts from 3387 and 1625 to 3416 and 1632 cm^−1^, respectively. Furthermore, the relative intensity of these bands above after As(V) sorption also had variation, these results illustrated the adsorption of As(V) may be related to the -OH group. Additionally, it can be seen from the spectrum that the peak at 862 cm^−1^ could be ascribed to the characteristic peak of v(As-O) bond, and some similar FTIR results about As adsorption have been reported by Goldberg^24^ and Suarez *et al*.^25^, so above results demonstrated that As(V) has been successfully adsorbed on the adsorbent iMNP. For the spectrum of cMNP, there are some changes about adsorption bands similar to that of iMNP. The being of ν(As—O), the variation of position and intensity of these bands in the spectrum of As loaded adsorbents confirmed the mechanism in Fig. 8.

### Adsorption kinetics

**Figure 9:**
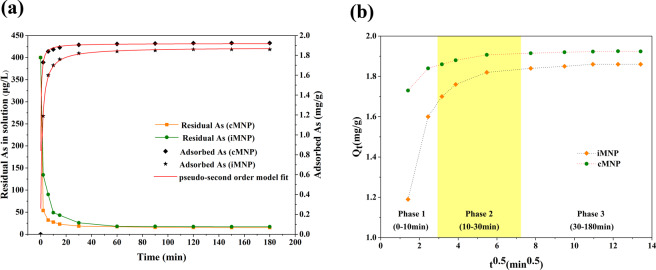
(**a**) Adsorption kinetics of As (V) on iMNP and cMNP. (**b**) As (V) adsorption modeling of kinetics data with Weber-Morris intraparticle diffusion plots. Adsorbents dose of 0.2 g/L, initial As (V) concentration of 400 ppb, temperature 25 °C, initial pH 6.6 ± 0.1, contact time 3 h.

The adsorption kinetics of As(V) by iMNP and cMNP were illustrated in Fig. 9a. As can be seen, both adsorbents had similar adsorption kinetics of As(V), it reached equilibrium within 60 min with over 90% of As (V) removed in 3 h for the initial As(V) concentration of 400 ppb. To better describe the adsorption kinetics of As(V), pseudo-first order kinetic model and the pseudo-second order kinetic model (Nonlinear Eqs. (3) and (4), respectively) were used to evaluate the removal kinetics of As(V) on the iMNP and cMNP^11^.

3 $${{\rm{q}}}_{{\rm{t}}}={q}_{e}(1-{e}^{-{k}_{1}t})$$

4 $${{\rm{q}}}_{{\rm{t}}}={\left(\frac{1}{{q}_{e}},+,\frac{1}{{k}_{2}{q}_{e}^{2}},{t}^{-1}\right)}^{-1}$$

where q_e_ and q_t_ are the amount of adsorbed As(V) (mg/g) at equilibrium and at time t respectively, k_1_ (min^−1^) and k_2_ (g/(mg·min) are the rate constants of pseudo-first order and pseudo-second order respectively. It can be seen from the kinetics parameters of both adsorbents presented in Table 2 that the coefficient of determination of pseudo-first order kinetics r^2^(iMNP) and r^2^(cMNP) are 0.9863 and 0.9973 respectively, and that of pseudo-second order kinetics r^2^(iMNP) and r^2^(cMNP) are 0.9996 and 0.9997. Furthermore, for both adsorbents, the values of q_e,calc_ derived from the pseudo-second order kinetic model were closer to the values of q_e,exp_ than that of the pseudo-first order kinetic model. Therefore, the removal kinetics of As(V) by the prepared adsorbents could be described well with the pseudo-second order kinetic model, which indicates the chemisorption occurred between As(V) and the adsorbents with the form of sharing or exchange of electrons might be the rate controlling process of As(V) adsorption^26^. Xie *et al*.^27^ assumed in the pseudo-second order process, the adsorption is via surface reaction until all the surface sites were occupied, subsequently the diffusion occurs at the adsorbents for further complexation interactions.

**Table 2:** Parameters of the adsorption kinetic models.

adsorbent	Q_e,exp_(mg/g)	Pseudo-first order kinetic model			pseudo-second order kinetic model		
		Q_e,calc_(mg/g)	k_1_(min^−1^)	r^2^	Q_e,calc_(mg/g)	k_2_(g·mg^−1^·min^−1^)	r^2^
iMNP	1.854	1.811	0.4891	0.9863	1.879	0.4753	0.9996
cMNP	1.921	1.899	1.2036	0.9973	1.920	2.2538	0.9997

To further investigate the removal process of As(V) by the fabricated materials, the Weber−Morris intraparticle pore diffusion model was used to examines the kinetic data. The model’s representation is presented in Eq. (5).

5 $${Q}_{t}=k{t}^{0.5}+c$$

Where Q_t_ (mg/g) is the amount of As(V) adsorbed by the adsorbents at time t (min), k (mg/(g·min^0.5^)) is the intraparticle diffusion rate constant, and c (mg/g) is the intercept that represents the boundary layer effect. With the larger the value of intercept c, the boundary layer effect (film diffusion) become more intense, which implies the contribution of the surface adsorption increase in the rate limiting steps. If the relationship between Q_t_ and t_0.5_ is linear and the intercept is zero, the intraparticle diffusion could be the only adsorption rate-controlling process^27^. In the intraparticle diffusion plot (Fig. 9b), one can observe that the liner plot of the both fabricated adsorbents didn’t pass through the origin, and the intercept of cMNP is larger than that of iMNP, which indicates adsorption process were controlled by intraparticle diffusion and the boundary layer effect^28,29^, and for cMNP, the effect of the film diffusion in the rate limiting process is greater than that of iMNP. Additionally, the observed multilinearity in the intraparticle diffusion plot was found in some previous studies^30,31^, they reported the multiple nature implied the adsorption process followed two or more phase. As shown in the Fig. 9b, the initial and instantaneous adsorption between arsenic and surface of the adsorbents as the first phase, where the amount of As adsorbed of iMNP is less than that of cMNP, which may be due to a little surface adsorption sites was occupied by some impurities, the second phase is the arsenic species gradually diffuse into the pores of γ-Fe_2_O_3_, where the intraparticle diffusion is the dominate rate-limiting step, the final phase is the adsorption equilibrium attribute to the chemical reaction/bonding.

### Adsorption isotherms

The adsorption isotherms of iMNP and cMNP for As(V) were investigated to determine the adsorption capacity, and the Langmuir and Freundlich isotherm models were used to analyze the experimental data.

Freundlich isotherm assumes that there are heterogeneous adsorption sites on the surface of the adsorbent, and the more binding sites occupied, the more difficult the adsorption, and multilayer adsorption may occur. The nonlinear form of Freundlich isotherm^32^ can be expressed as:

6 $${{\rm{q}}}_{{\rm{e}}}=k{c}_{e}^{1/n}$$

where q_e_ (mg/g) is the amount As(V) adsorbed per unit mass of adsorbent, c_e_ (mg/L) is the concentration of As(V) at the equilibrium, k indicates the affinity of adsorbent towards As(V), and n denotes the adsorption intensity.

**Figure 10:**
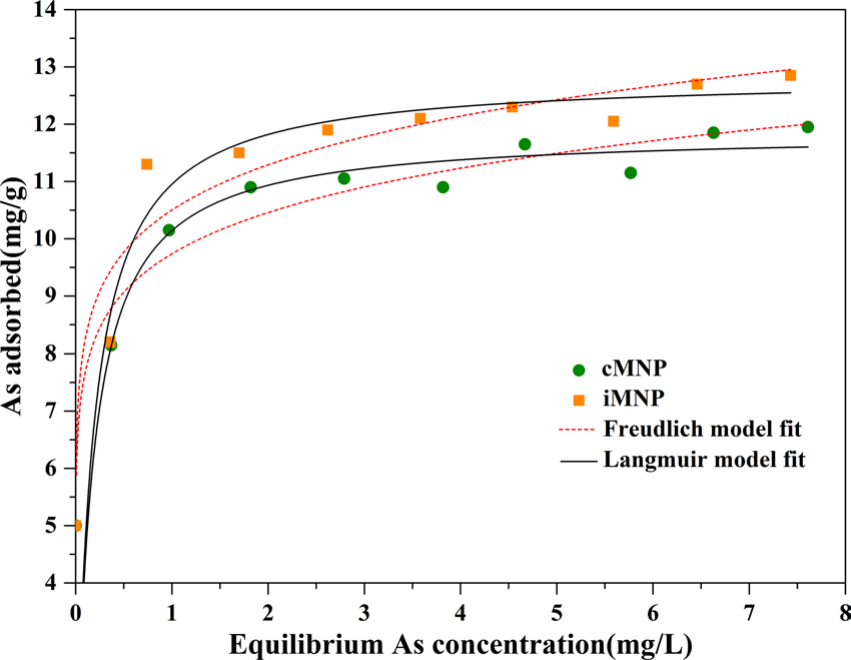
As (V) adsorption isotherms of iMNP and cMNP at 25°C, initial solution pH 6.6 ± 0.1, adsorbents dose 0.2 g/L, contact time 12 h.

Langmuir isotherm equation is derived from the assumption that monolayer adsorption can occur on the surface of the adsorbent, where the equivalent binding sites number is specific and no adsorbate transmigrate^32^. The Langmuir isotherm can be given in the following form:

7 $${{\rm{q}}}_{e}=\frac{{q}_{m}b{c}_{e}}{1+b{c}_{e}}$$

where q_m_ (mg/g) is the maximum adsorption capacity of the adsorbent and b (L/mg) is the Langmuir affinity constant, which is related to the energy of adsorption. The results and the parameters of the model fitting were displayed in Fig. 10 and Table 3, respectively. Comparing the coefficients of determination (R^2^) derived from the two adsorption isotherms, either for iMNP (R^2^_Lan_ = 0.8976, R^2^_Fre_ = 0.7236) or the cMNP (R^2^_Lan_ = 0.9378, R^2^_Fre_ = 0.8217), the Langmuir isotherm model explains the behaviour of As(V) adsorbed by the adsorbents better than the Freundlich isotherm model. The maximum adsorption capacity (q_m_) of iMNP (12.74 mg/g) for As(V) at pH 6.6 is slightly larger than that of cMNP (11.76 mg/g), which may be attributed to the impurities in no-crystalline forms, it is known that the amorphous is beneficial to adsorption process by increase the surface area of adsorbent, which was agreement with the Brunauer-Emmett -Teller analysis results that the surface area of iMNP (145.5 m^2^/g) is larger than that of cMNP (70.3 m^2^/g). The adsorption capacity of both adsorbents in this study were compared with those of other adsorbents (Table 4). It can be seen that iMNP’s adsorption capacity for As(V) was lower than unmodified WTRs and some iron-based adsorbents modified by biochars, chitosan or other materials. However, it is worth mentioning that, compared with some magnetic adsorbents also prepared with WTRs, the adsorption capacity of iMNP is more commendable. In addition, maghemite(γ-Fe_2_O_3_) nanoparticles reported previously^18,33^ could all maintained high arsenic adsorption efficiency throughout consecutive regeneration cycles, so the iMNP mainly consisted of γ-Fe_2_O_3_ in this study should have similar regeneration mechanism, but the regeneration effect would be affected by impurities. The constant n, a parameter of the Freundlich model, can represent the adsorption characteristics, for the n < 1, the adsorption is very difficult, when n values between 1 and 2 the adsorption is moderately difficult, when the values of n is between 2 and 10, the adsorption is easy to occur^10^. The n values of adsorbents prepared our studies are 9.556 and 9.703 correspond to iMNP and cMNP respectively, suggesting the removal of As(V) by both adsorbents are practical.

**Table 3:** Parameters of the Freundlich and Langmuir isotherm model.

	Freundlich isotherm			Langmuir isotherm		
	K	1/n	R^2^	q_m_(mg/g)	b(L/mg)	R^2^
iMNP	10.50	0.100	0.7236	12.74	6.0250	0.8976
cMNP	9.7445	0.097	0.8217	11.76	6.1692	0.9378

**Table 4:** Comparison of adsorption capacities for As(V) (Langmuir model) on both sorbents versus previously reported adsorbents.

Adsorbent	Maximum adsorption capacity for As(V) (mg/g)	pH	T(°C)	Ref.
Water treatment residuals (Fe/Al/Mn)	3.3–50	7.2	23	^34^
WTRs-loaded alginate beads	2.86	4.5	25	^35^
Magnetic particles prepared with WTRs	8.694	7.0	25	^18^
iron impregnated biosorbents	2.0	7.0	25	^36^
Waste Fe-Mn oxides embedded in chitosan	26.8	6.5	25	^37^
MIL-100(Fe)/rGO/δ-MnO_2_	162.07	2.0	45	^38^
Hybrid bamboo biochar Fe_3_O_4_ sorbents	868.0	7.0	40	^39^
iMNP	12.74	6.6	25	This research
cMNP	11.76	6.6	25	This research

## Conclusion

Employing the simple co-precipitation method, iron-containing water treatment residuals produced from drinking-water treatment plant were used to prepare magnetic nanoparticles (iMNP), which was used to compare with magnetic nanoparticles (cMNP) prepared with chemical agents. The results of this study are as follows: (i) The magnetic nanoparticles, iMNP and cMNP, consist of γ-Fe_2_O_3_ and their saturation magnetization are 35.5 emu/g and 69.0 emu/g, respectively. Consequently, both adsorbents could be easily separated and recycled from solutions with a hand held magnet; (ii) For both adsorbents, the As(V) adsorption isotherms fit the Langmuir adsorption isotherm model better than Freundlich isotherm model, indicating the adsorption of As(V) on the surface of the adsorbents is monolayer adsorption. Furthermore, the maximum adsorption capacity of iMNP (12.74 mg/g) is slightly larger than that of cMNP (11.76 mg/g); (iii) The pseudo-second order model could better described the adsorption kinetics of both adsorbents, which could remove over 90% of As(V) (400 μg/L) from water at pH 6.6 with the dosage of 0.2 g/L. In conclusion, the iMNP synthesized in our study is a kind of cost-effective and environment friendly substitute for cMNP.

## References

[1] Xi CY (2020). The fabrication and arsenic removal performance of cellulose nanocrystal-containing absorbents based on the “bridge joint” effect of iron ions. Carbohyd Polym..

[2] Hamza MF (2020). As(V) sorption from aqueous solutions using quaternized algal/polyethyleneimine composite beads. Sci Total Environ..

[3] Haque MA, Chowdhury RA, Islam S, Bhuiyan MS, Ragib AB (2020). Sustainability assessment of arsenic-iron bearing groundwater treatment soil mixed mortar in developing countries, bangladesh. J Environ Manage..

[4] Bessaies H (2020). Synthesis of novel adsorbent by intercalation of biopolymer in LDH for the removal of arsenic from synthetic and natural water. J Environ Sci..

[5] Ohta M, Okawa H, Kato T, Sugawara K (2020). Removal of arsenite from aqueous solutions using ultrasonic irradiation in the presence of a lead electrode. Jpn J Appl Phys..

[6] Parihar P (2015). Arsenic contamination, consequences and remediation techniques: A review. Ecotox Environ Safe..

[7] Guo J (2019). Loading NiCo alloy nanoparticles onto nanocarbon for electrocatalytic conversion of arsenite into arsenate. Electrochem Commun..

[8] Gupta VK, Carrott PJM, Ribeiro Carrott MML, Suhas (2009). Low-cost adsorbents: growing approach to wastewater treatment—a review. Crit Rev Env Sci Tec..

[9] Kurniawan TA, Sillanpää MET, Sillanpää M (2012). Nanoadsorbents for remediation of aquatic environment: local and practical solutions for global water pollution problems. Crit Rev Env Sci Tec..

[10] Jiang W (2013). Chromium(VI) removal by maghemite nanoparticles. Chem Eng J..

[11] Shan C, Tong M (2013). Efficient removal of trace arsenite through oxidation and adsorption by magnetic nanoparticles modified with Fe–Mn binary oxide. Water Res..

[12] Rashid M, Sterbinsky G, Gracia E (2018). Kinetic and mechanistic evaluation of inorganic arsenic species adsorption onto humic acid grafted magnetite nanoparticles. J Phys. Chem. C..

[13] Zhao Q, Doherty LP, Doyle D (2011). Fate of water treatment residual: an entire profile of Ireland regarding beneficial reuse. Int J Environ Stud..

[14] Makris KC, O’Connor GA (2007). Chapter 28 Beneficial utilization of drinking-water treatment residuals as contaminant-mitigating agents. Developments in Environmental Science..

[15] Zhu S (2015). A novel conversion of the groundwater treatment sludge to magnetic particles for the adsorption of methylene blue. J Hazard Mater..

[16] Elkhatib EA (2018). Using nanoparticles from water treatment residuals to reduce the mobility and phytoavailability of Cd and Pb in biosolid-amended soils. Environ Geochem Health..

[17] Chiang YW (2012). Adsorption of multi-heavy metals onto water treatment residuals: sorption capacities and applications. Chem Eng J..

[18] Zeng H (2018). As(V) Removal from water using a novel magnetic particle adsorbent prepared with iron-containing water treatment residuals. ACS Sustainable Chem. Eng..

[19] Yuan L, Weng X, Xie J, Du W, Deng L (2013). Solvothermal synthesis and visible/infrared optical properties of Al/Fe_3_O_4_ core–shell magnetic composite pigments. J Alloy Compd..

[20] Ma Z, Guan Y, Liu H (2005). Synthesis and characterization of micron-sized monodisperse superparamagnetic polymer particles with amino groups. J Polym Sci Pol Chem..

[21] Aronniemi M, Sainio J, Lahtinen J (2005). Chemical state quantification of iron and chromium oxides using xps: the effect of the background subtraction method. Surf Sci..

[22] Gota S, Guiot E, Henriot M, Gautiersoyer M (1999). Atomic-oxygen-assisted MBE growth of α-Fe_2_O_3_ on α-Al_2_O_3_(0001): metastable FeO(111)-like phase at subnanometer thicknesses. Phys.rev.b..

[23] Li P, Jiang EY, Bai HL (2011). Fabrication of ultrathin epitaxial γ-Fe_2_O_3_ films by reactive sputtering. J. Phys. D: Appl. Phys..

[24] Goldberg S, Johnston CT (2001). Mechanisms of arsenic adsorption on amorphous oxides evaluated using macroscopic measurements, vibrational spectroscopy, and surface complexation modeling. J.Colloid Interface Sci..

[25] Suarez DL, Goldberg S, Su C (1998). Evaluation of oxyanion adsorption mechanisms on oxides using FTIR spectroscopy and electrophoretic mobility. Am. Chem. Soc. Symp. Ser..

[26] Skumryev V (2003). Beating the superparamagnetic limit with exchange bias. Nature..

[27] Xie F (2014). Removal of phosphate from eutrophic lakes through adsorption by *in situ* formation of magnesium hydroxide from diatomite. Environ Scie Technol..

[28] Hamayun M (2013). Equilibrium and kinetics studies of arsenate adsorption by FePO_4_. Chemosphere..

[29] Hameed BH, Salman JM, Ahmad AL (2009). Adsorption isotherm and kinetic modeling of 2,4-D pesticide on activated carbon derived from date stones. J Hazard Mater..

[30] Rashid M, Price NT (2017). Gracia Pinilla, Miguel ángel, & O“Shea, K. E. Effective removal of phosphate from aqueous solution using humic acid coated magnetite nanoparticles. Water Res..

[31] Singh SK, Townsend TG, Mazyck D, Boyer TH (2012). Equilibrium and intra-particle diffusion of stabilized landfill leachate onto micro- and meso-porous activated carbon. Water Res..

[32] Reed BE, Matsumoto MR (1993). Modeling cadmium adsorption by activated carbon using the langmuir and freundlich isotherm expressions. Sep Sci Technol..

[33] Tuutijärvi T, Vahalaa R, Sillanpitää M, Chen G (2012). Maghemite nanoparticles for As(V) removal: desorption characteristics and adsorbent recovery. Environ Technol..

[34] Elkhatib E, Mahdy A, Sherif F, Hamadeen H (2015). Evaluation of a novel water treatment residual nanoparticles as a sorbent for arsenic removal. J. Nanomater..

[35] Ociński D, Jacukowicz-Sobala I, Kociołek-Balawejder E (2016). Alginate beads containing water treatment residuals for arsenic removal from water—formation and adsorption studies. Environ. Sci. Pollut. R..

[36] Verma L, Siddique MA, Singh J, Bharagava RN (2019). As(III) and As(V) removal by using iron impregnated biosorbents derived from waste biomass of Citrus limmeta (peel and pulp) from the aqueous solution and ground water. J Environ Manage..

[37] Ociński D, Mazur P (2020). Highly efficient arsenic sorbent based on residual from water deironing –Sorption mechanisms and column studies. J Hazard Mater..

[38] Ploychompoo S (2020). Fast and efficient aqueous arsenic removal by functionalized MIL-100(Fe)/rGO/δ-MnO_2_ ternary composites: Adsorption performance and mechanism. J Environ Sci..

[39] Alchouron J (2020). Assessing south american guadua chacoensis bamboo biochar and Fe_3_O_4_ nanoparticle dispersed analogues for aqueous arsenic(V) remediation. Sci Total Environ..

